# Arterial Baroreflex Inhibits Muscle Metaboreflex Induced Increases in Effective Arterial Elastance: Implications for Ventricular-Vascular Coupling

**DOI:** 10.3389/fphys.2022.841076

**Published:** 2022-03-25

**Authors:** Joseph Mannozzi, Jong-Kyung Kim, Javier A. Sala-Mercado, Mohamed-Hussein Al-Hassan, Beruk Lessanework, Alberto Alvarez, Louis Massoud, Tauheed Bhatti, Kamel Aoun, Donal S. O’Leary

**Affiliations:** Department of Physiology, Wayne State University School of Medicine, Detroit, MI, United States

**Keywords:** effective arterial elastance (Ea), arterial baroreflex, muscle metaboreflex activation, ventricular vascular coupling, neural control of cardiovascular system

## Abstract

The ventricular-vascular relationship assesses the efficacy of energy transferred from the left ventricle to the systemic circulation and is quantified as the ratio of effective arterial elastance to maximal left ventricular elastance. This relationship is maintained during exercise via reflex increases in cardiovascular performance raising both arterial and ventricular elastance in parallel. These changes are, in part, due to reflexes engendered by activation of metabosensitive skeletal muscle afferents—termed the muscle metaboreflex. However, in heart failure, ventricular-vascular uncoupling is apparent and muscle metaboreflex activation worsens this relationship through enhanced systemic vasoconstriction markedly increasing effective arterial elastance which is unaccompanied by substantial increases in ventricular function. This enhanced arterial vasoconstriction is, in part, due to significant reductions in cardiac performance induced by heart failure causing over—stimulation of the metaboreflex due to under perfusion of active skeletal muscle, but also as a result of reduced baroreflex buffering of the muscle metaboreflex-induced peripheral sympatho-activation. To what extent the arterial baroreflex modifies the metaboreflex-induced changes in effective arterial elastance is unknown. We investigated in chronically instrumented conscious canines if removal of baroreflex input via sino-aortic baroreceptor denervation (SAD) would significantly enhance effective arterial elastance in normal animals and whether this would be amplified after induction of heart failure. We observed that effective arterial elastance (E_a_), was significantly increased during muscle metaboreflex activation after SAD (0.4 ± 0.1 mmHg/mL to 1.4 ± 0.3 mmHg/mL). In heart failure, metaboreflex activation caused exaggerated increases in E_a_ and in this setting, SAD significantly increased the rise in E_a_ elicited by muscle metaboreflex activation (1.3 ± 0.3 mmHg/mL to 2.3 ± 0.3 mmHg/mL). Thus, we conclude that the arterial baroreflex does buffer muscle metaboreflex induced increases in E_a_ and this buffering likely has effects on the ventricular-vascular coupling.

## Introduction

During prolonged exercise the metabolite balance in active skeletal muscle becomes skewed as a result of an inability to actively remove metabolic waste. These excess metabolites in turn activate metabosensitive skeletal muscle afferents that enhance sympathetic outflow to the heart and peripheral vasculature—termed the muscle metaboreflex (Kaufman et al., 1983, 1984; Sunagawa et al., 1983; Adreani et al., 1985; Stone et al., 1985; Hansen et al., 1994; Boushel et al., 1998; Sala-Mercado et al., 2007; Kaur et al., 2016). The increased sympathetic outflow to the heart induces profound increases in ventricular function and heart rate, which thereby increases cardiac output (Melcher and Donald, 1981; O’Leary et al., 1997, 1999; Augustyniak et al., 2000; Ansorge et al., 2002; Crisafulli et al., 2003, 2006; Sala-Mercado et al., 2014; Mannozzi et al., 2021b). Indeed, during submaximal dynamic exercise increases in cardiac output are the primary mechanism mediating metaboreflex induced increases in arterial pressure as little net peripheral vasoconstriction usually occurs (Hanna and Kaufman, 1985; Sheriff et al., 1990; Kelly et al., 1992; O’Leary et al., 1997, 1999; Crisafulli et al., 2003, 2011; Sala-Mercado et al., 2014; Mannozzi et al., 2021b). Muscle metaboreflex vascular control is restrained via reflex induced β_2_ mediated vasodilation, as well as selective arterial baroreflex buffering of peripheral sympatho-activation which combined limits increases in systemic vasoconstriction (Kelly et al., 1992; Kim et al., 2005b; Ky et al., 2013). However, in heart failure the mechanisms mediating muscle metaboreflex pressor responses shifts as a result of an inability to improve ventricular function and cardiac output, not only due to the overarching pathology but also due to profound coronary vasoconstriction which actively restrains increases in cardiac function (Melcher and Donald, 1981; O’Leary et al., 1985; Ansorge et al., 2005; Sala-Mercado et al., 2006; Crisafulli et al., 2007; Coutsos et al., 2013; Mannozzi et al., 2021b). Muscle metaboreflex activation in heart failure now elicits substantial increases in peripheral sympathetic activity that evokes vasoconstriction of even the active skeletal muscle—the very tissue from which the reflex arises (Hanna and Kaufman, 1985; Rotto and Kaufman, 1985; Hammond et al., 2001; Kaur et al., 2017). The exaggerated vascular responses observed in heart failure are, in part, a result of attenuated arterial baroreflex buffering of the muscle metaboreflex (Kim et al., 2005b; Ky et al., 2013). In heart failure the strength of the arterial baroreflex is reduced thereby limiting the ability to buffer metaboreflex—induced sympatho-activation (Chen et al., 1991; Osterziel et al., 1995; Fisher et al., 2005; Kim et al., 2005a,b; Iellamo et al., 2006; Otsuki et al., 2008; Kaur et al., 2015a). These profound changes in arterial baroreflex—muscle metaboreflex interactions in heart failure could have marked effects on effective arterial elastance and thereby impact total systemic perfusion by impeding ventricular—arterial energy transfer.

Ventricular—vascular coupling is an assessment of cardiovascular efficiency wherein energy transfer to and propagation of that energy through the systemic circulation is quantified by assessing changes in arterial and ventricular elastance (Chantler et al., 1985; Burkhoff and Sagawa, 1986; Little and Cheng, 1991; Sved et al., 2000; Loimaala et al., 2003; Kim et al., 2011). These metrics were first developed in part as a mechanism to deduce stroke work (Sunagawa et al., 1985; Thames et al., 1993), and then latter uses and modifications of each metric were used to ascertain aspects of ventricular function (Wyss et al., 1983; Cote et al., 1985; O’Leary, 1985; Burkhoff and Sagawa, 1986; Little and Cheng, 1991; Robert et al., 2000; Sved et al., 2000; Loimaala et al., 2003; Sala-Mercado et al., 2006, 2014; Sheriff, 2006) and the arterial load imposed on the left ventricle—termed effective arterial elastance (E_*a*_) (Chantler et al., 1985; Burkhoff and Sagawa, 1986; Kim et al., 2011; Reddy et al., 2017; Mannozzi et al., 2021b). The latter of these two metrics coupled with measurements of stroke work has been used to deduce the relative maintenance of ventricular-vascular coupling at rest and during exercise in animal models and humans (Kaufman et al., 1982; Chantler et al., 1985; O’Leary, 1985; Burkhoff and Sagawa, 1986; Kass, 2005; Redfield et al., 2005; Chantler and Lakatta, 2012; Reddy et al., 2017; Mannozzi et al., 2021b). Most notably in healthy active subjects alterations in E_*a*_ contribute to the maintenance of the ventricular-vascular coupling relationship and that even with additional sympathetic input during exercise, such as with muscle metaboreflex activation, the relative changes in E_*a*_ favor improvements in stroke work (Melcher and Donald, 1981; Sala-Mercado et al., 2014; Mannozzi et al., 2020, 2021b). However, in heart failure the ventricular-vascular relationship is significantly uncoupled at rest and this relationship unravels further with reflex sympathetic activation as increases in E_a_ remain unmatched due to an inability to improve ventricular function and thus the ability to improve stroke work is stifled (Melcher and Donald, 1981; Sala-Mercado et al., 2006; Mannozzi et al., 2021b).

The significant increases in E_a_ observed during sympathetic activation during exercise in normal and heart failure subjects are a result of large increases in heart rate but also though significant increases in peripheral vasoconstriction (Mannozzi et al., 2021b), both of which are not only affected by muscle metaboreflex activation but also likely modified by arterial baroreflex function during exercise (Augustyniak et al., 1985; Hanna and Kaufman, 1985; O’Leary and Augustyniak, 1998; Farquhar et al., 2000; Hammond et al., 2001; Ichinose et al., 2002; Kim et al., 2005a,b; Iellamo et al., 2006; Ky et al., 2013; Kaur et al., 2015a,b, 2018; Jozwiak et al., 2019; Mannozzi et al., 2021b). To date, no study has evaluated the relative contribution of arterial baroreflex buffering of muscle metaboreflex induced increases on E_a_ and stroke work. Furthermore, the relative assessment of these variables also pertains to the level of control the arterial baroreflex exerts that inhibits or engenders muscle metaboreflex maintenance of ventricular-vascular coupling during exercise. We hypothesize that the arterial baroreflex likely restrains muscle metaboreflex induced increases in E_a_. In heart failure, arterial baroreflex function is significantly attenuated and thus the ability to restrain increases in E_a_ will be reduced and could thereby contribute to enhanced ventricular-vascular uncoupling observed during muscle metaboreflex activation in heart failure.

## Materials and Methods

### Experimental Subjects

10 mongrel canines of either sex of 18–25 kg were selected based on their willingness to run on a motor driven treadmill at 3.2 km/h with 0% grade. We have previously shown that gender does not influence the strength or mechanisms of muscle metaboreflex activation (Limberg et al., 2015). Additionally, studies in young adults have shown that gender does not significantly impact baroreflex mediated responses (Furlan et al., 1998; Kim et al., 2004). All animals in this study underwent a 14-day acclimatization to laboratory spaces and personnel prior to any volitional exercise and surgical procedures. Five of the 11 animals were selected to undergo sino-aortic barodenervation via two separate anesthetic events after completion of control experiments. All surgical and experimental protocols outlined in this study were reviewed and approved by the Wayne State University Institute for the Care and Use Committee (IUCAC) and meet requirements set by the National Institutes of Health Guide to the Care and Use of Laboratory Animals.

### Surgical Instrumentation

All animals utilized in this study underwent a series of two surgical procedures with a minimum of 14 days to recover between procedures. The anesthetic and analgesic regimen for all surgical procedures is as follows; animals were initially anesthetized using Thiopental sodium (25 mg/kg) and maintained using isoflurane gas (1.5–2.5%). Analgesia was maintained by application of a fentanyl patch (TD 125–150 μg/h) preoperatively and left for 3 days. To prevent infection prophylactic antibiotics Cephazolin (500 mg IV) was administered during the procedure and Cephalexin (30 mg/kg) was administered after twice daily by mouth. Additional analgesic management was provided during the recovery from each procedure by administration of buprenorphine (0.015 mg/kg IV) and acepromazine (0.1 mg/kg IM) on an as needed basis. All anesthetic, analgesic and surgical procedures performed in this study have been used previously (Hanna and Kaufman, 1985; Augustyniak et al., 2000; Hammond et al., 2001; Kim et al., 2005a,b; Sala-Mercado et al., 2006, 2014; Ky et al., 2013).

The first surgical procedure was a left thoracotomy in which a 20PAU flow probe (Transonic Systems, Ithaca NY) was placed on the ascending aorta for measures of cardiac output and 3 stainless steel pacing electrodes were sutured to the apex of the left ventricle for induction of heart failure via rapid ventricular pacing at 225–245 bpm for 25–30 days. In this procedure unrelated to the current study two sonomicrometry crystals (Ontario, Canada) were implanted into the myocardium, a 3PSB flow probe (Transonic Systems, Ithaca NY) was placed on the circumflex artery. The pericardium was reapproximated and the chest was closed in layers. All cables and leads were exteriorized dorsally between the scapulae. Animals were given a minimum of 14 days to recover prior to the next procedure.

The second surgical procedure utilized a left retroperitoneal approach for placement of a 10PAU blood flow probe (Transonic Systems, Ithaca, NY) on the terminal aorta to measure hindlimb blood flow. Caudally to the flow probe a vascular occluder (Invivo Metric, Healdsburg CA) was placed to induce progressive reductions in hindlimb blood flow to elicit the muscle metaboreflex during exercise. Cranially to the flow probe a 19-gauge polyvinyl catheter (Tygon, S54-HL, Norton) was placed for measures of mean arterial pressure. Unrelated to the current study a 4PSB flow probe (Transonic Systems, Ithaca, NY) was placed on the renal artery. Additionally, 7 animals in this study during this procedure were instrumented with a 19-gauge polyvinyl catheter (Tygon, S54-HL, Norton) in the jugular vein for measures unrelated to the current study. The surgical site was closed in layers and all cables, catheters and occluder lines were tunneled subcutaneously and exteriorized dorsally between the scapulae. Animals were given 14 days minimally to recover prior to any experimental protocols.

*Sino-Aortic Baroreceptor Denervation (SAD)* consisted of two additional anesthetic events in five animals after completion of control experimental protocols utilizing the same anesthetic and analgesic methods described above. Removal of sino-aortic baroreceptor input was achieved through bilateral transection of the aortic depressor and carotid sinus nerves as previously described by Sheriff et al. (1987), Kim et al. (2005b), and Ky et al. (2013). Animals were given a minimum of 7 days to recover prior to transection of the contralateral side of the previous procedure. All five animals received 7 days minimally after the second transection to recovery prior to any experimental procedures. Functional assessments of denervation were ascertained via lack of any significant change in heart rate in response to increases in arterial pressure (> 30 mmHg) induced i.v., infusion of phenylephrine.

*Placement of a Central Venous Catheter* was performed during the second procedure in animals which did not undergo SAD by placing a catheter in the jugular vein and advancing it to the atrial caval junction. In subjects that underwent SAD the CVP catheter was placed during the SAD procedure in the same manner as the control animals. The central venous catheter was used to assess central venous pressure.

### Experimental Procedures and Data Acquisition

All experiments were performed minimally 14 days after the second surgical procedure or minimally 7 days after the second SAD procedure. All animals served as their own control within their separate experimental groups. These groups were divided as follows: 5 animals performed control experiments prior to immediate induction of heart failure via rapid ventricular pacing (Control_group_). The second group also contained 5 animals which after completion of control experiments underwent SAD and then repeated all the control experiments prior to induction of heart failure (SAD_group_). For each experiment animals were brought to the laboratory space and acclimated to the environment and personnel for 15–30 min prior to the beginning of the experiment. Once the experiment began the animals flow probes were connected to bench top flow meters (Transonic Systems, Ithaca NY) and catheters were connected to pressure transducers (Transpac IV; Abbott Laboratories, Abbot Park IL). The pressure transducers were connected to a Gould recording system model RS3800 (Dataq, Akron Ohio). All data was acquired in Windaq (Dataq, Akron Ohio) acquisition software. Heart rate was determined using a cardiotachometer that was triggered by the peak cardiac output signal. One-minute steady state values were taken during rest, exercise (3.2 km/h with 0% grade), and during exercise (3.2 km/h with 0% grade) with sequential reductions in hindlimb blood flow to elicit the muscle metaboreflex in a controlled repeatable fashion using the hindlimb vascular occluder. These experiments were repeated after induction of heart failure via rapid ventricular pacing at a rate of 225–245 bpm for 25–30 days in the Control_group_ animals. In the SAD_group_ animals, experiments were repeated after SAD prior to heart failure induction and then repeated after.

### Metaboreflex Activation

The muscle metaboreflex is a sympathetically mediated response elicited by buildup of metabolic waste in active skeletal muscle (Kaufman et al., 1983, 1984; Mittelstadt et al., 1985; Stone et al., 1985; Boushel et al., 1998; Hammond et al., 2001; Kaur et al., 2016). This reflex was first discovered as a result of circulatory occlusion of exercising voluntary muscles in humans (Alam, 1937) which continues to be used as a technique to investigate this reflex across a variety of models (White, 1981; Iellamo et al., 1997; Hammond et al., 2000; Ichinose et al., 2002, 2017; Crisafulli et al., 2003, 2006, 2007, 2011; Edwards et al., 2008; Fisher et al., 2010; Hart et al., 2010; Choi et al., 2012, 2013; Antunes-Correa et al., 2014; Boulton et al., 2018; Cristina-Oliveira et al., 2020). Studies in canines show that the muscle metaboreflex is not tonically active at low workloads inasmuch as hindlimb blood flow must be reduced below a critical threshold level before reflex responses are observed (Hanna and Kaufman, 1985; Rotto and Kaufman, 1985; Kelly et al., 1992; O’Leary et al., 1997; Augustyniak et al., 2000; Hammond et al., 2001; Kaur et al., 2015b,^2017^; Mannozzi et al., 2020). As workload increases, this threshold becomes closer and closer to the normal prevailing level of blood flow such that at relatively moderate workloads no threshold is often observed indicating that this reflex is tonically active (Hanna and Kaufman, 1985; Hammond et al., 2001). After induction of heart failure, during moderate exercise hindlimb blood flow is well below the threshold as ascribed in control experiments. Thus, indicating that at least a portion of the excessive sympatho-activation seen during moderate exercise in heart failure is due to excessive activation of the muscle metaboreflex (Hanna and Kaufman, 1985; Kelly et al., 1992; Augustyniak et al., 2000; Hammond et al., 2001; Kaur et al., 2015b). Thus, the use of artificially induced metabolite accumulation in active skeletal muscle via partial reductions in blood flow affords a repeatable, reproducible manner in which to understand muscle metaboreflex characteristics with and without disease.

### Data Analysis

One-minute steady state averages of cardiac output, heart rate, mean arterial pressure, hindlimb blood flow, and other data unrelated to this study was exported into excel and calculations of additional hemodynamic parameters were completed. Steady state averages were taken after a 3-min acclimatization period at each workload, rest, exercise, and exercise with each reduction in hindlimb blood flow.

### Calculations

Stroke Volume = Cardiac output/heart rate

Non-Ischemic Vascular Resistance (NIVR)—an assessment of the resistance of the entire vascular system that subtracts the contribution of the ischemic hindlimb.

NIVR = mean arterial pressure/(cardiac output – hindlimb blood flow)

Effective Arterial Elastance (E_a_) = NIVR × heart rate

Stroke Work = (Stroke Volume/1,000) × mean arterial pressure

### Statistics

Systat Software (Systat 13.0) was used for statistical analyses. Data were analyzed using a Two-way ANOVA for repeated measures within each group (Control_group_, SAD_group_) where each animal in each group served as its own control. No direct assessments were performed across groups comparing Control_group_ to SAD_group_ except for assessing the relative change between normal heart failure and heart failure with SAD. When a significant interaction was observed, individual means were compared using a C matrix test for simple effects and a modified Bonferroni was used as a *post-hoc* test. The relative change in one variable between two states within the same group was assessed using a Students Paired *t*-test after determining the variables normality using a Shapiro-Wilks test. For the assessment of relative change between normal heart failure and heart failure with SAD a Welches *t*-test was used after determining the variables normality using a Shapiro-Wilks test. All data are reported as means ± standard error of the mean. Statistical significance was determined by an α level of *P* < 0.05.

## Results

**FIGURE 1:**
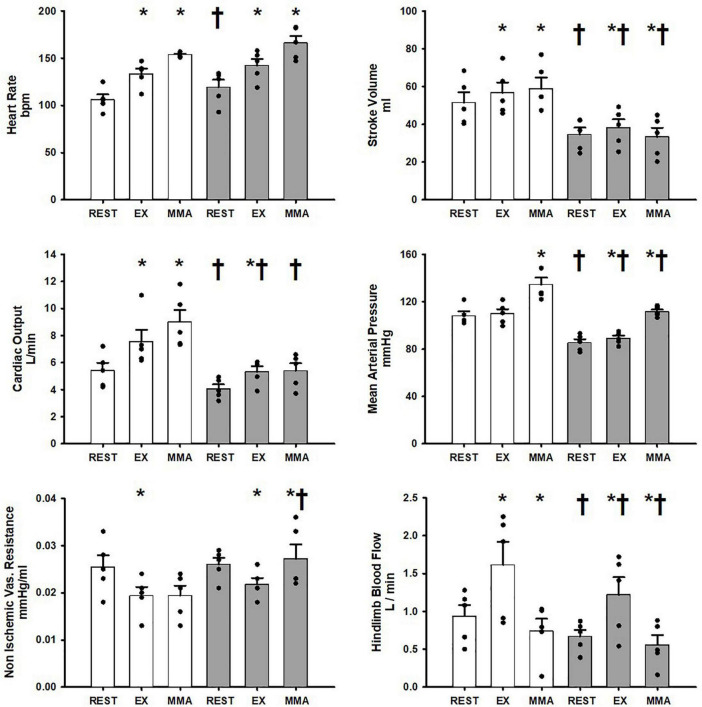
Average 1-min steady state hemodynamics from the Control_group_ at rest, exercise (EX), and exercise with muscle metaboreflex activation (MMA) before (white) and after induction of heart failure (gray). Data are reported as means with standard error. Observed data points are overlain on corresponding bar graphs. Statistical significance vs. previous workload is depicted as * and vs. previous condition as ^†^ where *P* < 0.05. (*N* = 5).

Figure 1 shows the average hemodynamic responses in the Control_group_ at rest, exercise, and exercise with muscle metaboreflex activation (MMA) before and after induction of heart failure. In healthy subjects during the transition from rest to exercise significant increases in heart rate, stroke volume, cardiac output, and hindlimb blood flow occurred. No significant change in mean arterial pressure occurred during the transition from rest to exercise. Non-ischemic vascular resistance was significantly reduced with exercise. In response to muscle metaboreflex activation, heart rate, stroke volume, cardiac output, and mean arterial pressure all significantly increased whereas, no significant change in non-ischemic vascular resistance occurred. Hindlimb blood flow was significantly reduced with muscle metaboreflex activation as a result of inflation of the hindlimb vascular occluders. At rest after induction of heart failure, stroke volume, cardiac output, mean arterial pressure and hindlimb blood flow were all significantly lower than the control values. During steady state mild exercise after the induction of heart failure, heart rate, stroke volume, cardiac output, mean arterial pressure and hindlimb blood flow all increased vs. the values observed at rest. Non ischemic vascular resistance was significantly reduced in the transition from rest to exercise. Although, significant increases in stroke volume, cardiac output, mean arterial pressure and hindlimb blood flow occurred with exercise in heart failure, all of these values were significantly reduced vs. control. No significant changes as a result of heart failure induction were observed in heart rate, and non-ischemic vascular resistance during exercise. Hindlimb blood flow was significantly reduced in heart failure during muscle metaboreflex activation when compared to control. Muscle metaboreflex activation after induction of heart failure caused significant increases in heart rate and mean arterial pressure whereas stroke volume was significantly reduced. Non-Ischemic vascular resistance increased significantly with Muscle metaboreflex activation in heart failure and was significantly greater when compared to control. After induction of heart failure, muscle metaboreflex activation failed to induce any significant increase in cardiac output which remained significantly lower than the corresponding value in control. Furthermore, during muscle metaboreflex activation in heart mean arterial pressure and stroke volume were reduced relative to control.

**FIGURE 2:**
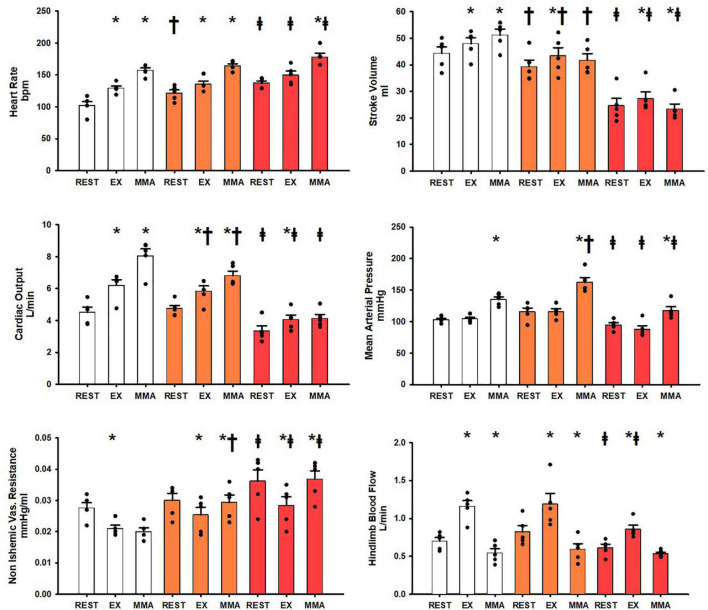
Average 1-min steady state hemodynamics from the SAD_group_ at rest, exercise (EX), and exercise with muscle metaboreflex activation (MMA) before (white) an after SAD (orange) and after induction of heart failure post SAD (red). Data are reported as means with standard error. Observed data points are overlain on corresponding bar graphs. Statistical significance vs. previous workload depicted as * where *P* < 0.05. Comparisons of the condition of SAD vs. control depicted as ^†^ where *P* < 0.05. Comparisons of the condition of heart failure vs. SAD depicted as ^‡^ where *P* < 0.05. (*N* = 5).

Figure 2 shows the hemodynamic responses in the SAD_group_ during rest, exercise, and muscle metaboreflex activation. All of the control hemodynamics of the SAD_group_ from rest to exercise and exercise to muscle metaboreflex activation show the same significant changes as from the Control_group_ (Control data for both Control_group_ and SAD_group_ shown in white in both Figures 1, 2). After SAD (orange bars) all resting baseline values were the same except for a significant increase in heart rate and a significant reduction in stroke volume. After SAD significant increases in heart rate, stroke volume, cardiac output, and hindlimb blood flow occurred with exercise similarly as in the Control_group_. A significant reduction in non-ischemic vascular resistance was observed, while no significant change was observed in mean arterial pressure with exercise. SAD significantly reduced cardiac output and stroke volume during exercise relative to control. With muscle metaboreflex activation after SAD, significant increases in heart rate, cardiac output, mean arterial pressure, and non-ischemic vascular conductance were observed. No significant change was observed in stroke volume. SAD induced significant reductions in stroke volume and cardiac output during exercise and muscle metaboreflex activation relative to control. A significant increase in non-ischemic vascular resistance was observed during muscle metaboreflex activation as a result of SAD relative to control. Heart failure (red) induction induced significant reductions in stroke volume, cardiac output, mean arterial pressure, and hindlimb blood flow, in the SAD_group_ baseline resting values. Conversely significant increases in heart rate, and non-ischemic vascular resistance were observed at rest after induction of heart failure relative to SAD (orange). In response to exercise after induction of heart failure in the SAD_group_, significant increases in stroke work, cardiac output, and hindlimb blood flow occurred. No significant change was observed in heart rate, and significant reductions in non-ischemic vascular resistance were observed. Heart failure induced significant reductions in stroke volume, cardiac output, mean arterial pressure, and hindlimb blood flow relative to control. Non-ischemic vascular resistance was significantly increased during exercise after induction of heart failure relative to control. In the transition from exercise to muscle metaboreflex activation in heart failure significant increases in heart rate, mean arterial pressure, and non-ischemic vascular resistance occurred. Conversely a significant reduction in stroke volume occurred while no significant increase in cardiac output occurred in response to muscle metaboreflex activation during heart failure. Heart failure induction after SAD induced significant reductions in stroke volume, mean arterial pressure, and cardiac output during muscle metaboreflex activation whereas heart failure induced significant increases in non-ischemic vascular resistance and heart rate. There was no significant difference between hindlimb blood flow during metaboreflex activation in SAD or SAD after induction of heart failure.

**FIGURE 3:**
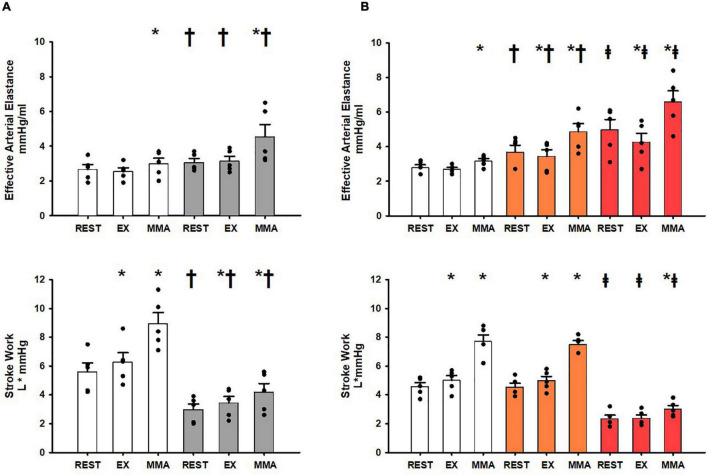
**(A)** Average 1-min steady state values of Effective Arterial Elastance and Stroke Work in the Control_group_ at rest, exercise (EX), and exercise with muscle metaboreflex activation (MMA) before (white) and after induction of heart failure (gray). Statistical significance vs. previous workload depicted * and vs. previous condition as ^†^ where *P* < 0.05. (*N* = 5). **(B)** Average 1-min steady state values of Effective Arterial Elastance and Stroke Work in the SAD_group_ at res, exercise (EX), and exercise with muscle metaboreflex activation (MMA) before (white) and after SAD (orange), and after heart failure induction post SAD (red). Statistical significance vs. previous workload depicted as * where *P* < 0.05. Comparisons of the condition of SAD vs. control depicted as ^†^ where *P* < 0.05. Comparisons of the condition of heart failure vs. SAD depicted as ^‡^ where *P* < 0.05. (*N* = 5) Data for **(A,B)** are reported as means with standard error. Observed data points are overlain on corresponding bar graphs.

Figure 3A shows the changes in a component (E_a_) and an index (stroke work) of ventricular-vascular coupling at rest, exercise, and muscle metaboreflex activation in both the Control_group_ before (white) and after induction of heart failure (gray). In response to mild exercise no significant changes occurred in E_a_ whereas stroke work was significantly increased. With muscle metaboreflex activation significant increases in stroke work and E_a_ occurred albeit the increase in E_a_ was small but this is likely a result of this conservative assessment of E_a_ as shown previously by Mannozzi et al. (2020, 2021b). After induction of heart failure significant increases in E_*a*_ were only in response to muscle metaboreflex activation. E_a_ during rest, exercise, and muscle metaboreflex activation was significantly increased relative to control. Stroke Work was significantly lower in rest, exercise, and muscle metaboreflex activation after induction of heart failure, however, significant increases in the responses from rest to exercise and exercise to muscle metaboreflex activation were observed. Figure 3B shows the changes in E_a_ and stroke work in the SAD_group_ before (white) and after SAD (orange) as well as with SAD after induction of heart failure (red). Responses in E_a_ and stroke work before SAD in normal animals were similar to the Control_group_. After SAD (orange) Ea was significantly increased at all workloads relative to control whereas stroke work was not significantly different at rest or during exercise or muscle metaboreflex activation relative to SAD_group_ control. Significant increases were observed between exercise and muscle metaboreflex activation in both E_*a*_ and stroke work, furthermore the increase in E_a_ was significantly greater compared to SAD_*group*_ control. After induction of heart failure (red) E_a_ was significantly enhanced, and stroke work was significantly attenuated at all workloads relative to control. In response to exercise E_a_ was significantly reduced and no significant change was observed in stroke work. With muscle metaboreflex activation in heart failure, significant increases in E_*a*_ and stroke work occurred, however, the level of stroke work observed during muscle metaboreflex activation was significantly attenuated and the level of E_a_ was significantly increases relative to SAD (orange).

**FIGURE 4:**
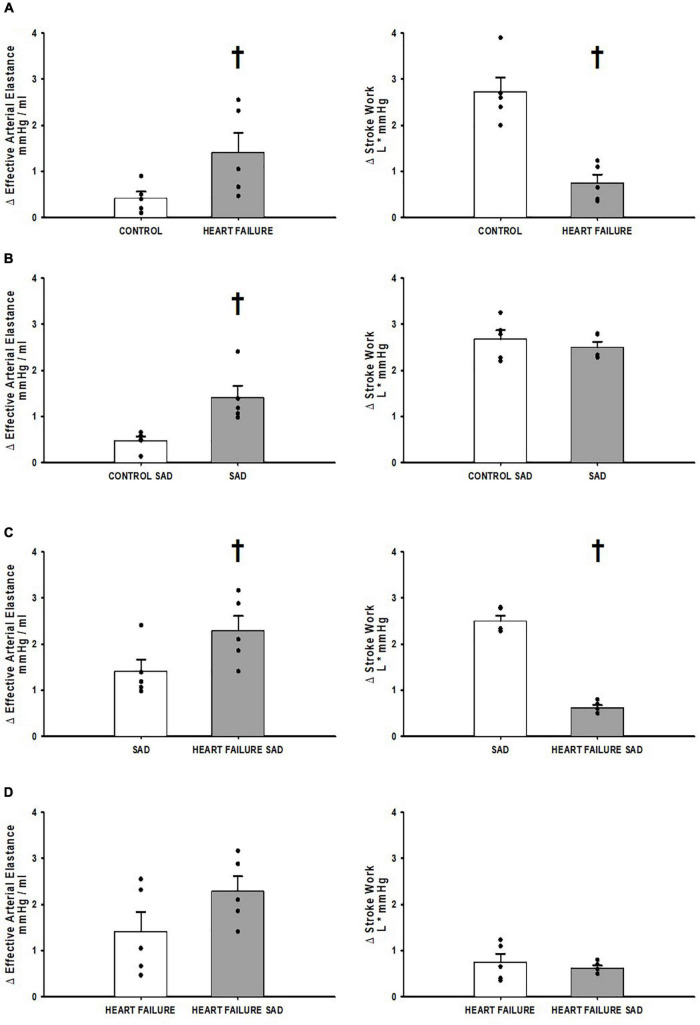
**(A)** The relative change in Effective Arterial Elastance and Stroke Work in the transition from exercise to exercise with muscle metaboreflex activation before (white) and after induction of heart failure (gray) in the Control_group_. **(B)** The relative change in Effective Arterial Elastance and Stroke Work in the transition from exercise to exercise with muscle metaboreflex activation before (white) and after SAD (gray) in the SAD_group_. **(C)** The relative change in Effective Arterial Elastance and Stroke Work in the transition from exercise to exercise with muscle metaboreflex activation in normal animals with SAD (white) and after induction of heart failure post SAD (gray) in the SAD_group_. **(D)** The relative change in Effective Arterial Elastance and Stroke Work in the transition from exercise to exercise with muscle metaboreflex activation in heart failure animals from the Control_group_ (white) and heart failure animals post SAD from the SAD_group_ (gray). For all panels data are reported as means with standard error where data points are overlain on corresponding bar graphs. Statistical significance vs. the previous state as ^†^where *P* < 0.05. An *N* = 5 was used for each bar graph in every panel.

Figure 4 shows the relative change from exercise to muscle metaboreflex activation in the assessments of ventricular-vascular coupling; E_a_ and stroke work. Figure 4A shows the relative change in control and heart failure in the Control_group_ where the change in E_a_ was significantly increased and the change in stroke work was significantly attenuated in heart failure. Figure 4B shows the relative change before and after SAD. SAD caused a significant increase in the relative change of E_a_ from exercise to muscle metaboreflex activation with no significant change in stroke work. Figure 4C shows the relative change in the SAD_group_ between SAD prior to and after induction of heart failure. Significant increases in the relative change of E_a_ were observed in the heart failure group relative to control. The relative change in stroke work was significantly less in heart failure when compared to control. Figure 4D is a comparison of the relative change in heart failure from the Control_group_ and the SAD_group_. This assessment was done using a Welches *t*-test for assumed unequal variances due to the different treatment of SAD. The SAD heart failure E_a_ response was larger but not significantly different from control. Furthermore, no significant relative change from exercise to muscle metaboreflex activation in stroke work was observed.

**FIGURE 5:**
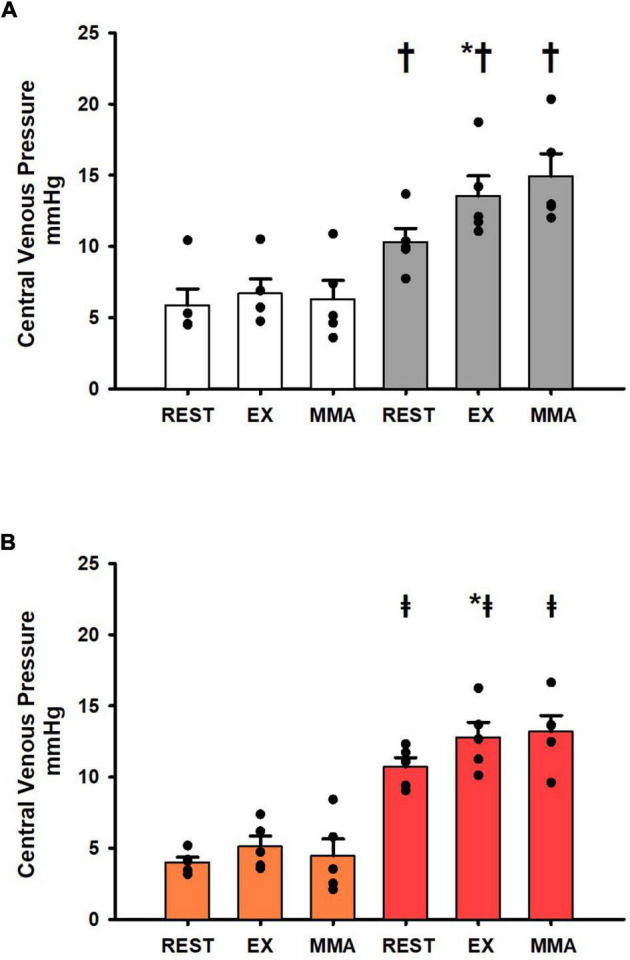
**(A)** Average 1-min steady state values of central venous pressure from the Control_group_ at rest, exercise (EX), and exercise with muscle metaboreflex activation (MMA) before (white) and after induction of heart failure (gray). Data are reported as means with standard error. Observed data points are overlain on corresponding bar graphs. Statistical significance vs. previous workload is depicted as * and vs. previous condition as ^†^ where *P* < 0.05. (*N* = 5). **(B)** Average 1-min steady state values of central venous pressure from the SAD_group_ at rest, exercise (EX), and exercise with muscle metaboreflex activation (MMA) after SAD (orange) and after induction of heart failure post SAD (red). Data are reported as means with standard error. Observed data points are overlain on corresponding bar graphs. Statistical significance vs. previous workload depicted as * where *P* < 0.05. Comparisons of the condition of SAD vs. Heart failure SAD depicted as ^‡^ where *P* < 0.05. (*N* = 5).

Figure 5 shows the changes in central venous pressure at rest, exercise, and exercise with muscle metaboreflex activation in both the Control_group_ and SAD_group_ before and after induction of heart failure. No significant changes were observed in central venous pressure across rest, exercise, and exercise with muscle metaboreflex activation before induction of heart failure (Figure 5A). After induction of heart failure central venous pressure was significantly increased across all settings. Furthermore, central venous pressure significantly increased from rest to exercise, however, there was no change in CVP with metaboreflex activation. These changes in CVP with exercise and metaboreflex activation before and after induction of heart failure were similar after SAD (Figure 5B).

## Discussion

This is the first study to quantify arterial baroreflex restraint of muscle metaboreflex-induced increases in effective arterial elastance and how this interaction changes after the induction of heart failure. We observed that the arterial baroreflex significantly buffers muscle metaboreflex—induced increases in E_a_ likely via restraint of peripheral vasoconstriction. This buffering could contribute importantly to the ability to raise cardiac output inasmuch as large increases in E_*a*_ act to inhibit effective energy transfer from the heart to the vasculature. Despite the large increases in E_*a*_ with metaboreflex activation in normal animals after SAD, stroke work was well maintained. This is likely due to large increases in ventricular contractility as we have previously shown that in baro-intact animals metaboreflex activation causes substantial increases in ventricular maximal elastance (O’Leary et al., 1985, 1999; Sala-Mercado et al., 2006, 2014). Furthermore, we observed that in heart failure, E_a_ is significantly enhanced as previously described (Mannozzi et al., 2021b). The rise in E_a_ with metaboreflex activation after SAD in heart failure tended to be greater (*p* = 0.07, Figure 4D) suggesting that although arterial baroreflex buffering capacity in heart failure is attenuated, some degree of restraint may be maintained.

### Arterial Baroreflex—Muscle Metaboreflex Interaction

The arterial baroreflex is the primary mechanism maintaining homeostatic blood pressure through postural changes and exercise (Mitchell et al., 1983; Ferguson et al., 1992; Farquhar et al., 2000; DiCarlo and Bishop, 2001; Ichinose et al., 2002, 2008; Iellamo et al., 2006, 2013; Joyner, 2006; Edwards et al., 2008; Greaney et al., 2014; Kaur et al., 2015a; Jozwiak et al., 2019). Moreover, the arterial baroreflex has been previously described as a buffer for muscle metaboreflex induced pressor responses during exercise (Sheriff et al., 1987; Ichinose et al., 2002, 2008, 2015; Kim et al., 2005a,b; Iellamo et al., 2006, 2013; Joyner, 2006; Edwards et al., 2008; Fisher et al., 2010; Ky et al., 2013; Kaur et al., 2015a,^2018^; Fu and Ogoh, 2019). The arterial baroreflex modulates pressure through dynamic control of both the parasympathetic and sympathetic arms of the autonomic system. First and foremost, baroreflex function modulates parasympathetic activity to induce rapid changes in heart rate to modify cardiac output and therefore blood pressure (Vianna et al., 1985; Little and Cheng, 1993; Osterziel et al., 1995; Segers et al., 2002; Iellamo et al., 2006; Joyner, 2006; Harthmann et al., 2007; Edwards et al., 2008; Chen et al., 2010; Walley, 2016; Al-Khateeb et al., 2017). Secondarily, the arterial baroreflex modulates sympathetic activity to the heart and peripheral vasculature to maintain blood pressure (Mitchell et al., 1983; Ferguson et al., 1992; Sheriff et al., 1998; Farquhar et al., 2000; DiCarlo and Bishop, 2001; Zucker, 2006; Greaney et al., 2014; Walley, 2016; Jozwiak et al., 2019). The latter of these two mechanisms of blood pressure regulation is likely the primary mechanism by which the arterial baroreflex alters the muscle metaboreflex induced pressor responses during exercise (Kim et al., 2005b; Ky et al., 2013).

The muscle metaboreflex induces profound increases in blood pressure during exercise primarily through robust increases in ventricular maximal elastance and heart rate which thereby increases cardiac output and blood pressure (Melcher and Donald, 1981; Drew et al., 1985; Rotto and Kaufman, 1985; Olivier and Stephenson, 1993; O’Leary et al., 1999; Augustyniak et al., 2000; Crisafulli et al., 2003, 2006, 2011; Boushel, 2010; Mannozzi et al., 2021b). The metaboreflex also exerts control over the peripheral vasculature by both eliciting paradoxical β_2_ mediated peripheral vasodilation via release of epinephrine from the adrenal glands and counteracting sympathetic vasoconstriction of inactive vascular beds as well as the coronary circulation and even the active skeletal muscle (Coutsos et al., 2010, 2013; Hanna and Kaufman, 1985; Augustyniak et al., 2000; Hammond et al., 2001; Najjar et al., 2004; Ansorge et al., 2005; Kaur et al., 2015b,^2017^, ^2018^). The net results are a little marked change in total peripheral resistance which is reflected by only small increases in E_*a*_. When left unchecked by the arterial baroreflex, the muscle metaboreflex is capable of marked peripheral vasoconstriction including within the active skeletal muscle itself which thereby amplifies the metaboreflex responses causing large increases in E_a_ (Kelly et al., 1992; Kim et al., 2005b; Ky et al., 2013; Kaur et al., 2015a,b, 2017). Therefore, baroreflex restraint of metaboreflex pressor responses occurs primarily via inhibition of peripheral vasoconstriction. Thus, the much greater metaboreflex pressor response after SAD stems from substantial systemic vasoconstriction now joining the large increases in cardiac output which then can produce profound increases in arterial blood pressure (Sheriff et al., 1987; Kim et al., 2005b; Ky et al., 2013).

In heart failure, the ability to raise ventricular function during metaboreflex activation is markedly impaired, both due to the inherent ventricular dysfunction as well as heightened reflex coronary vasoconstriction (Coutsos et al., 2010, 2013; Hanna and Kaufman, 1985; Hammond et al., 2001; Ansorge et al., 2002, 2005). In contrast to normal individuals, in heart failure substantial peripheral vasoconstriction now occurs with metaboreflex activation (Najjar et al., 2004; Kim et al., 2005b; Crisafulli et al., 2007; Ky et al., 2013). This is likely due to impaired baroreflex buffering of peripheral sympatho-activation (Kim et al., 2005b; Ky et al., 2013). During metaboreflex activation in heart failure this enhanced systemic vasoconstriction coupled with increased tachycardia causes significantly greater increases in E_a_ from the already elevated levels. This shift in metaboreflex mechanisms toward peripheral vasoconstriction in heart failure remains after SAD, with a modest increase in the vasoconstrictor response and thus increases in E_a_. Thus, in contrast to the normal condition, SAD does not markedly alter the mechanisms of metaboreflex response, just allows some exaggeration of peripheral vasoconstriction which indicates a reduced role of the baroreflex in modifying the metaboreflex in heart failure. This is supported by the changes in E_a_ observed.

### Implications for Ventricular-Vascular Coupling

Ventricular-vascular coupling is a dynamic interplay between ventricular and vascular components ensuring adequate transfer and systemic propagation of ventricular work. Maintenance of this relationship is paramount to the ability to adequately provide systemic perfusion while enduring orthostatic postural changes and maintaining workload performance during exercise. A significant shift in ether the ability to maintain ventricular function, i.e., ventricular maximal elastance (Emax) or maintain an appropriate effective arterial elastance will ultimately upend this relationship, such as occurs in aging populations, heart failure, and hypertension (Kaufman et al., 1982; Chantler et al., 1985; Cote et al., 1985; O’Leary, 1985; Eichhorn et al., 1992; Laprad et al., 1999; Fadel, 2015; Chantler, 2017; Faconti et al., 2017) and thus may compromise the ability to maintain cardiovascular control during normal activities of daily life. These intolerances may be caused by both aberrant arterial baroreflex and muscle metaboreflex function.

**FIGURE 6:**
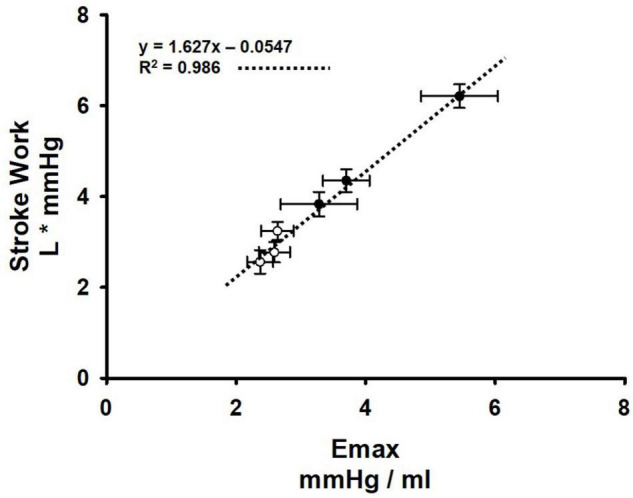
Relationship between ventricular maximal elastance (Emax) and stroke work before (black circles) and after induction of heart failure (white circles) at rest, mild exercise, and muscle metaboreflex activation. A linear regression (dotted line) was performed on these points. and the *R*_2_ value are shown on the plot. Error bars depict standard error of the mean in both directions (Data points were calculated from primary data collected in experiments which were reported in Coutsos et al. (2010, 2013) (*N* = 6).

In normal animals during muscle metaboreflex activation, we observed that little increase in Ea occurs and in previous studies we showed that Emax increases substantially with metaboreflex activation (Coutsos et al., 2010, 2013). Thus, the ventricular- vascular coupling ratio (E_a_/Emax) declines which favors the large increases in cardiac output and stroke work seen in the present and previous studies (Sala-Mercado et al., 2006, 2014; Mannozzi et al., 2021b). Stroke work can be affected by a number of factors including ventricular preload, afterload, and contractility. There were no effects of SAD or muscle metaboreflex activation on ventricular preload as reflected by the changes in CVP (Figure 5). Figure 6 shows primary data which were obtained in a previous study (Coutsos et al., 2013) now replotted anew describing the relationship between Emax and stroke work (*n* = 6). Shown are the average values at rest, mild exercise, and muscle metaboreflex activation before (closed circles) and after (open circles) the induction of heart failure. In these settings there was a markedly linear relationship between Emax and stroke work: as Emax rose with exercise and metaboreflex activation, highly proportional increases in stroke work also occurred (*R*^2^ = 0.986). In the present study or previous studies, we have not measured Emax after SAD, however we could calculate SW. In the normal animal after SAD, large increases in E_a_ occurred with similar increases in SW. Assuming the relationship shown in Figure 6 holds after SAD, this would indicate a similar increase in Emax occurred after SAD as previously shown in normal animals at this workload (Sala-Mercado et al., 2006, 2014). Thus, with a larger increase in E_*a*_ and little change in Emax, the ventricular—vascular relationship would become slightly less efficient thereby limiting the increase in cardiac output, as was observed (Figure 2). In heart failure, E_*a*_ is substantially increased at rest (Figure 1) and Emax is markedly attenuated (Sala-Mercado et al., 2006, 2014), thus the ventricular – vascular relationship is uncoupled causing a lower cardiac output (Sala-Mercado et al., 2006, 2014; Mannozzi et al., 2021b; Figure 2). In response to muscle metaboreflex activation, E_*a*_ increases substantially (Figure 1) however Emax is little changed (Sala-Mercado et al., 2006, 2014) which thereby further uncouples an already unbalanced ventricular—vascular relationship. After SAD, with metaboreflex activation the rise in E_a_ tended to be even more exaggerated (Figure 4, panel D, ΔE_*a*_ *p* = 0.07 heart failure vs. heart failure + SAD) and given the same small increase in stroke work, any small rise in Emax is likely little changed (Figure 6) from that observed in baro-intact animals (Sala-Mercado et al., 2006, 2014) therefore the substantially uncoupled ventricular-vascular relationship persists, preventing any increase in cardiac output.

### Limitations

We utilized imposed reductions in hindlimb blood flow to activate the muscle metaboreflex during mild exercise, a setting wherein little, if any, tonic activation of this reflex exists in normal subjects (Hanna and Kaufman, 1985; Augustyniak et al., 2001; O’Leary et al., 2004). However, in heart failure, hindlimb blood flow during exercise is low and often well below any threshold level necessary to activate the reflex. In this setting, metaboreflex responses are similar as those seen in normal subjects when hindlimb blood flow is reduced to similar levels (Hammond et al., 2001). So, whereas the methods we used artificially activate the metaboreflex, the responses observed likely reflect those seen with natural reflex stimulation. Recent studies in humans indicate that skeletal muscle afferents are activated at relatively low workloads and thus contribute to the normal cardiovascular responses to mild exercise and that this contribution is exaggerated in patients with heart failure (White, 1981; Amann et al., 1985, 2014; Barbosa et al., 2016).

In these studies, we measured CVP as an index of ventricular preload. However, CVP is not left ventricular end diastolic pressure. Thus, it is possible that changes in left ventricular preload did occur which could affect stroke work. However, Figure 6 shows a highly linear relationship between stroke work and Emax. spanning rest to exercise and metaboreflex activation before after induction of heart failure, therefore if preload changes did occur, it appears that the major factor affecting stroke work in these studies would be Emax and the resultant changes in ventricular-vascular coupling.

## Conclusion

We conclude that the arterial baroreflex actively restrains muscle metaboreflex induced increases in E_a._ This likely contributes to the ability of the muscle metaboreflex to preserve and even optimize the ventricular-vascular relationship through robust increases in ventricular elastance that are not overshadowed by effective arterial elastance (Melcher and Donald, 1981; Sala-Mercado et al., 2006, 2014; Mannozzi et al., 2021b). In heart failure, arterial baroreflex buffering of metaboreflex-induced sympatho-activation is reduced and thus contributes to an enhanced E_a_ during exercise. However, complete removal of the arterial baroreflex in heart failure reveled that a degree of restraint is intact likely preserving what little ventricular-vascular coupling remains. To what degree this restraint could be improved to further maintain or even restore some amount of ventricular-vascular coupling is unknown. Previous studies focused on the benefits of exercise regimes on the restoration of cardiac and autonomic function have shown promise in improving baroreflex function (Adreani et al., 1985; Andrade et al., 1985; Iellamo et al., 2007; Besnier et al., 2017; Mannozzi et al., 2021a) and this may be a mechanism of improving the ventricular-vascular coupling relationship in various cardiovascular pathologies.

## Data Availability Statement

The raw data supporting the conclusions of this article will be made available by the authors, without undue reservation.

## Ethics Statement

The animal study was reviewed and approved by the Wayne State University Institute for the Care and Use Committee (IUCAC).

## Author Contributions

DO’L and JM contributed to conception, design of research, performed the experiments, interpreted results of experiments, and drafted the manuscript. DO’L, JM, J-KK, M-HA-H, BL, AA, LM, TB, JS-M, and KA analyzed the data and performed the experiments. JM prepared the figures. DO’L, J-KK, M-HA-H, and JM edited and revised the manuscript. All authors approved final version of manuscript.

## Conflict of Interest

The authors declare that the research was conducted in the absence of any commercial or financial relationships that could be construed as a potential conflict of interest.

## Publisher’s Note

All claims expressed in this article are solely those of the authors and do not necessarily represent those of their affiliated organizations, or those of the publisher, the editors and the reviewers. Any product that may be evaluated in this article, or claim that may be made by its manufacturer, is not guaranteed or endorsed by the publisher.
